# Kinetic and analytical characterization of a new tropinone oxidase enzyme and its application to the simultaneous determination of the tropane alkaloids atropine and scopolamine

**DOI:** 10.1007/s00216-025-05856-6

**Published:** 2025-04-10

**Authors:** Mario Domínguez, Susana de Marcos, Javier Galbán

**Affiliations:** Analytical Chemistry Department, University of Zaragoza and, Instituto de Nanociencia y Materiales de Aragón (INMA), CSIC-Universidad de Zaragoza, 50009 Zaragoza, Spain

**Keywords:** NADH, Chia, Colorimetric enzymatic method, Michaelis–Menten kinetics

## Abstract

A spectrophotometric enzymatic method for the determination of atropine (Atp) and scopolamine (Scp), two tropane alkaloids (TAs), has been developed. The method is based on a previous basic hydrolysis to tropine (Trp) and scopine (Sci) respectively, and a subsequent enzymatic oxidation catalyzed by a tropinone reductase 1 (TRase) using NAD as oxidant; the absorbance of NADH (340 nm) is monitored during the reaction. First, the enzyme kinetics of both substrates are studied; it is concluded that both TAs follow a compulsory order ternary complex mechanism and the Michalis-Menten constant is calculated. Then, an enzymatic method was optimized for Atp, allowing the determination of this substrate in the range from 1.1·10^−5^ M to 3.0·10^−4^ M (LoD = 3.5·10^−6^ M); it was applied to the determination of Atp in a spiked chia sample (96 ± 6% recovery). Interestingly, the equilibrium constant of the reaction decreased with temperature and increased with enzyme concentration; both effects were satisfactorily explained. A similar analytical study was carried out with Scp (linear range from 1.2·10^−5^ M to 3.0·10^−4^ M, LoD = 3.6·10^−6^ M); the method was also applied to Scp in a spiked chia sample (94 ± 2% recovery). Finally, since the kinetics of Scp is slower than that of Atp, the simultaneous quantitative determination of both compounds was successfully developed by measuring the absorbance at two reaction times (70 s and 300 s). This method was applied to the simultaneous determination of both TAs first in a synthetic sample and later in a spiked chia sample, with recoveries around 98% for both compounds. Although the sensitivity of the method is lower than that of the immunoassays for Atp, it has advantages such as the simultaneous determination of Atp and Scp, and even the possible determination of Trp (another TA).

**Supplementary Information:**

The online version contains supplementary material available at 10.1007/s00216-025-05856-6.

## Introduction

**Fig. 1:**
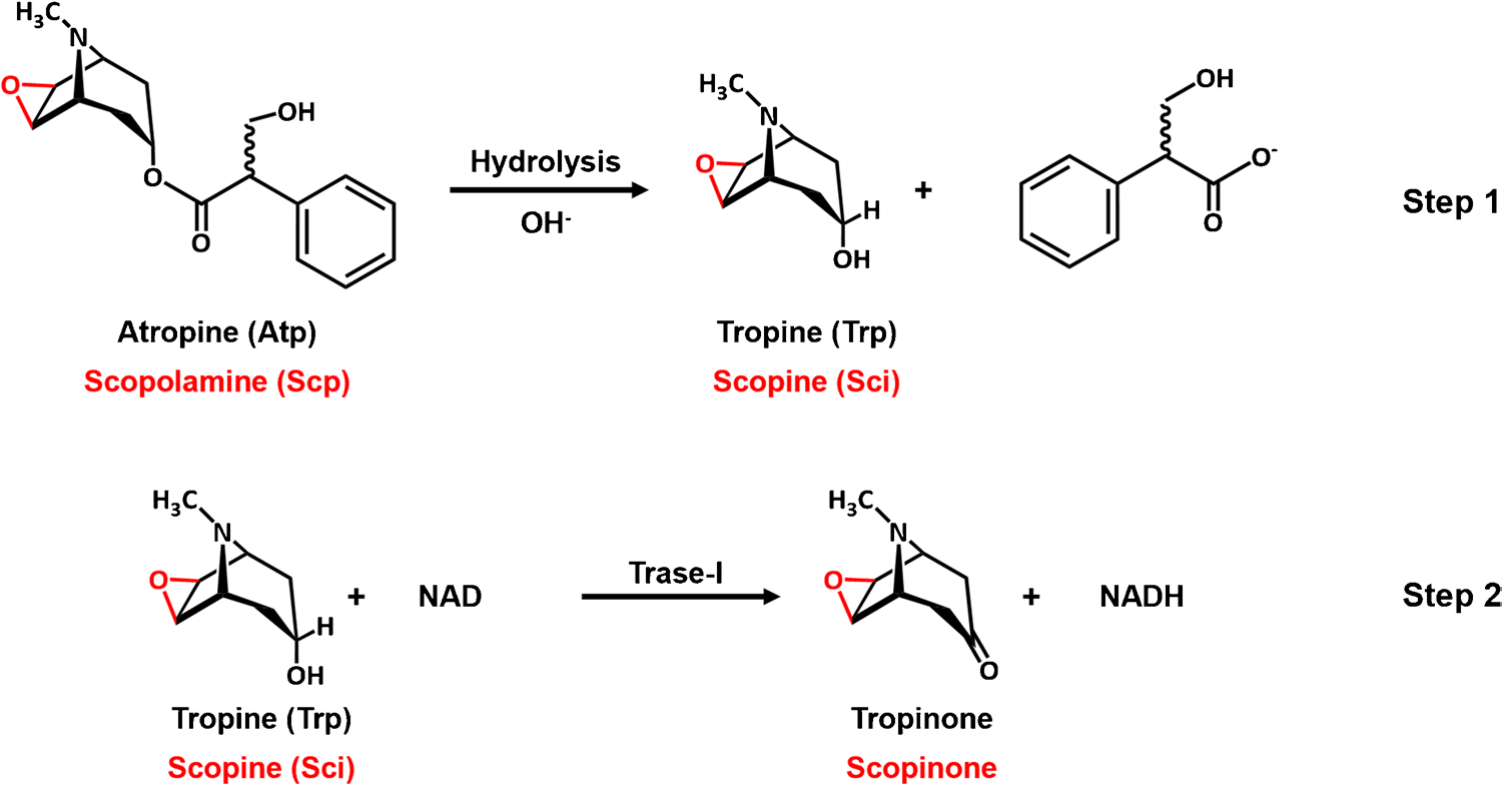
Schematic representation of the proposed analytical methodology

Tropane alkaloids (TA) are organic compounds with a nortropane structure (Fig. 1). These compounds are secondary metabolites synthesized by several plant families, especially the Solanaceae family (such as belladonna, henbane or stramonium), to defend them against pathogens and predators [1, 2]. More than 200 tropane alkaloids are known to occur in plants [3], but the most common is atropine (Atp) followed by scopolamine (Scp) (Fig. 1) [4, 5].

They have been extensively studied because of their use in the pharmaceutical industry, where they act as non-selective inhibitors of muscarinic acetylcholine receptors [6]. However, they can be highly toxic if the established dose is exceeded, causing various disorders in the human organism; hence, Scp is also known as burundanga as it is used illegally as a psychoactive drug. Because of the adverse health effects, the European Food Safety Authority (EFSA) has established an acute reference dose for Atp and Scp, or the EU has set maximum limits for Atp in several foods [7, 8]. The problem is that some types of food, especially cereals such as flax, soy, sorghum, millet, and sunflower, can contain high levels of TA due to seeds from the Solanaceae family of plants can contaminate them [9].

The analytical methods currently used for the determination of TA are based on the use of instrumental separation [10–12] and immunoassay techniques [13]. The use of liquid chromatography-tandem mass spectrometry (LC-MS2) has proven to be a highly sensitive technique for the identification of these metabolites, achieving detection limits below 5 ng/mL. Although these techniques provide optimal results, they are generally slow for quality control and require sample treatment such as derivatization. In addition, they are also generally expensive. For rapid screening and faster determination, there is a need to develop rapid (more than chromatography-based) and inexpensive (more than immunoassay-based) methods that allow an initial assessment of the toxicity of potentially harmful foods. Enzymatic methods combined with UV–vis molecular absorption are very well positioned due to their selectivity and robustness. This combination forms a group of analytical methods that are widely used for the determination of compounds of organic nature [14]. The applications of these methods range from the analysis of compounds of clinical interest to industrial analysis, food analysis or compounds of environmental interest [15]. However, to the best of our knowledge, only colorimetric methods without the selectivity offered by an enzymatic reaction have been proposed so far for the determination of tropane alkaloids [16], probably due to the lack of suitable enzymes. Furthermore, we found no references in the literature for the simultaneous determination of both analytes for non-instrumental separation-based methods.

To find suitable enzymatic reactions, we have set our sights on the synthetic routes of these compounds in plants [17, 18]. The complete biosynthetic pathway has not yet been clarified at all. It starts with the amino acids ornithine or arginine, and after several steps, tropinone is formed. This compound is enzymatically reduced by NAD(P)H, mediated by tropinone reductase I (TRase), to tropine (Trp), which then undergoes further enzymatic reactions to give Atp and finally Scp.

We have exploited the reversibility of the TRase reaction to develop analytical methods for Atp determination based on its previous chemical desterification (Fig. 1, step 1). In these methods, a new recombinant TRase is used, and the formed NAD(P)H is determined by in-situ formation of gold nanoparticles [19] or by coupling the classical formazan/diaphorase colorimetric method [20].

In this paper, we present a detailed study of the kinetics of this new TRase in relation to Atp and Scp by measuring the absorbance of NADH formed at 340 nm. The mechanism of the enzymatic reaction is elucidated; the Michaelis–Menten constants and the turnover number for both ATs are measured. Finally, these results have allowed us to develop an analytical method for the simultaneous determination of Atp and Scp.

## Materials and methods

### Reagents and solution

The following chemicals were used throughout the work: ß-nicotinamide adenine dinucleotide hydrate (NAD, Sigma-Aldrich N1511), atropine sulfate monohydrate (Sigma-Aldrich A0257), scopolamine hydrobromide (Sigma-Aldrich S0929), tropine (Sigma-Aldrich 93,550), scopine (Medchem Express HY-B0459A), recombinant tropinone reductase 1 (TRase; Gecco Biotech, EC 1.1.1.206), recombinant tropine esterase (TEase; Gecco Biotech, EC 3.1.1.10). All other reagents (such as buffer solutions) were of analytical grade and used without further purification.

### Apparatus

Agilent 8453A photodiode UV–vis spectrophotometer, SPECORD^R^ 210 Plus UV–vis molecular absorption spectrophotometer and Cary Eclipse fluorescence spectrophotometer (Agilent Technologies). The Millipore Milli-Q H_2_O system was used for water purification. The temperature of the reactions was controlled by a thermostatic bath connected to the cuvette compartment. Quartz, glass, and PMMA cells (1 cm pathlength) were used.

### Measurement procedure

Procedure for measuring tropine (Trp) and Scopine (Sci). Place 1935 μL 0.1 M HCO_3_^−^/CO_3_^2−^ buffer (pH 10) in the spectrophotometer cell, then add 40 μL 0.05 M NAD and 5 μL 8.8 mg/mL Trase (200 mM). Finally add 20 μL of the analyte (sample or standard solution) and start recording the absorbance at 340 nm. The initial reaction rate and the absorbance at equilibrium were used as the analytical parameters.

Procedure for the determination atropine (Atp) and scopolamine (Scp). First, hydrolysis was performed by placing in the cell 20 μL of the analyte solution (standard or sample) and 20 μL of 2 M NaOH solution and allowing to react for 5 min. Then, 1915 μL of 0.1 M NaHCO_3_/Na_2_CO_3_ buffer (pH 10), 40 μL 0.05 M NAD and 5 μL 8.8 mg/mL (200 mM) TRase were added (in the same cell) and the absorbance at 340 nm was monitored. The initial reaction rate and the absorbance at equilibrium were used as the analytical parameters.

For the determination of Atp and Scp in Chia sample, the solid was first spiked with both TA. After drying, the sample was treated according to the method proposed by Adamse [21]. Briefly, 4 g of sample was lixiviated with 40 mL methanol/water/formic acid (60/40/0.4, V/V) for 30 min. The suspension was then centrifugated and the supernatant subjected to the previously described procedure.

## Results and discussion

### Kinetic characterization of tropinone reductase

The TRase used in this paper was a recombinant enzyme (the gene was obtained from *Whitaria somnifera*). Prior to the development of the analytical method based on Fig. 1, the recombinant TRase used in this study was kinetically characterized for both hydrolysis products: tropine (Trp) and scopine (Sci).

Dehydrogenase enzymes normally follow a compulsory order ternary complex (COTC) kinetic mechanism (where NAD is the first substrate to be bound) [22] rather than a double-displacement (ping-pong) one. As this enzyme was developed for this study, the ping-pong mechanism was also considered. In both cases, the method based on the initial reaction rate was used.

First, the Trp kinetics were studied. For this purpose, a matrix of 36 experiments was prepared, using 6 concentration levels for NAD ranging from 5.0·10^−5^ M to 2.0·10^−3^ M and another 6 concentration levels for Trp ranging from 1.0·10^−5^ M to 6.0·10^−4^ M. The same concentration of TRase was added in all the experiments (0.50 mM), the temperature was kept at 25 ± 1 °C and the changes in the absorbance at 340 nm (maximum of the NADH absorption) were monitored. The detailed kinetic calculations are shown in supplementary material (Section S1). Table S1 shows the initial rates of the Abs = f(t) plots obtained for each of the 36 experiments, after discarding statistically anomalous data. The results were first fitted to the Lineweaver–Burk model to deduce whether the COTC (1) or ping-pong (2) mechanism was followed and to obtain a first estimate of the kinetic constants.

1 $$\frac{1}{{V}_{0}}=\left(\frac{{K}_{m,Trp}}{{k}_{cat}{[TRase]}_{0}}+\frac{{K}_{i,NAD}{K}_{m,Trp}}{{k}_{cat}{[TRase]}_{0}{[NAD]}_{0}}\right)\frac{1}{{[Trp]}_{0}}+\left(\frac{1}{{k}_{cat}{[TRase]}_{0}}+\frac{{K}_{m,NAD}}{{k}_{cat}{[TRase]}_{0}{[NAD]}_{0}}\right)$$

2 $$\frac{1}{{V}_{0}}=\left( \frac{{K}_{m,Trp}}{{k}_{cat}{[TRase]}_{0}}\right)\frac{1}{{\left[Trp\right]}_{0}} + \left(\frac{{K}_{m,NAD}}{{k}_{cat}{[TRase]}_{0}{\left[NAD\right]}_{0}}+\frac{1}{{k}_{cat}{[TRase]}_{0}}\right)$$

In these equations, K_m,Trp_ and K_m,NAD_ are the Michaelis–Menten constants for Trp and NAD, K_i,NAD_ is the inhibition constant for NAD, and k_cat_ is the corresponding catalytic constant for both models. According to these results (Fig. S1 and Table S2), the COTC mechanism was followed. The kinetic constants were then obtained more precisely using the conventional Michaelis–Menten equation for this type of mechanism (3) and the Solver™ routine of an Excel™ worksheet. The constants are given in Table 1 and the detailed calculation is explained in Section S1.3.

**Table 1:** Kinetic constants corresponding to the COTC mechanism for Trp and Sci

	K_m,NAD_,M	K_m,Trp_ or K_m,Scp_, M	K_i,NAD_, M	k_cat_, s^−1^
Tropine	1.6262(± 0.0004)·10^−3^	5.0(± 0.3)·10^−4^	4.7(± 0.6)·10^−4^	6.229 (± 0.005)·10^5^
Scopine	8.6(± 0.1)·10^−5^	5 (± 1)·10^−5^	3.3 (± 0.8)·10^−3^	1.391(± 0.002)·10^4^

3 $${V}_{0}=\frac{{k}_{cat}{[TRase]}_{0}\left[NAD\right]}{{K}_{a}\left[NAD\right]+{K}_{b}\left[Trp\right]+\left[Trp\right]\left[NAD\right]+{K}_{iA}{K}_{b}}$$

A similar scheme was used to investigate the kinetic mechanism of Sci. The detailed calculation is described in the supplementary material (Section S2). Briefly, a new matrix of 36 experiments was prepared and the initial rate was measured using the same procedure as for Trp, except that the working temperature was changed to 15 ± 1 °C (see below). From the results obtained, the COTC model was also followed, and a first estimate of the kinetic constants was made; these were then calculated more precisely (see Table 1).

Although the two series of constants were not calculated at the same temperature, the difference was not very great, and a certain type of comparison can be made. On the one hand, it is clear that the k_cat_ for Trp is about 45 times higher than that of Sci; this is very interesting from an analytical point of view as it allows the kinetic differentiation between the two compounds and the simultaneous determination of both analytes. On the other hand, it seems that Sci appears to have a greater affinity for TRase than Trp, but both K_m_ are statistically similar, so it can be concluded that the affinity of the enzyme for both substrates is similar.

### Development of a method for atropine (Atp) determination

#### Tropine (Trp) determination with Tropinone Reductase (TRase)

The optimal conditions for the determination of Atp were studied. Considering that the method involves a hydrolysis of Atp to Trp and a later Trp oxidation, both steps were optimized separately. For the reaction of Trp with TRase the optimum NAD concentration was considered 1.0·10^−3^ M (see previous section); pH and buffer nature, reaction temperature and enzyme concentration were studied. The Abs = f(t) recorder at 340 nm was obtained in all cases and the absorbance at the equilibrium (Abs_eq_) was used as the analytical parameter for a better sensitivity.

**Fig. 2:**
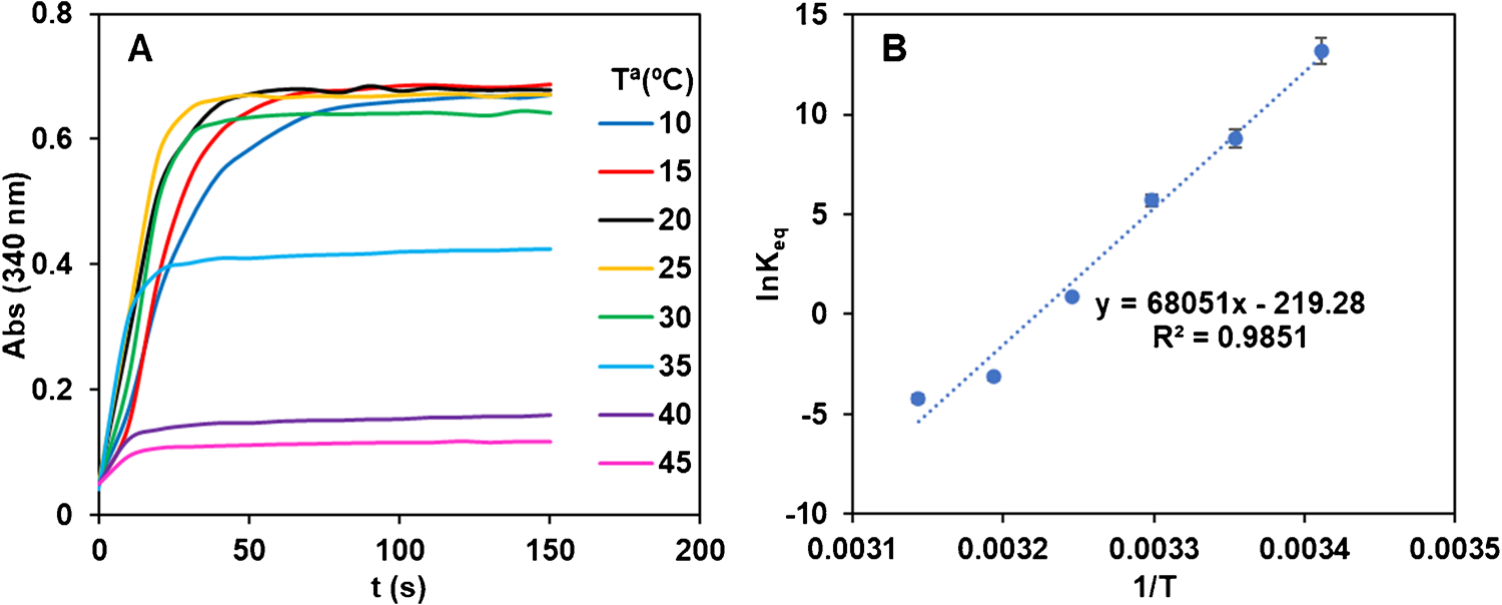
**A)** Abs=f(t) recordings obtained at different working temperatures. [NAD] =1.0·10^−3^ M, [Trp] = 1.0·10^−3^M, [TRase] = 0.5 mM, pH = 10 (HCO_3_^-^/CO_3_^2-^ 0.1 M), λ = 340 nm; **B)** Variation of the ln_Keq_=f(1/T) derived from values of figure A

As shown in Fig. S3, pH = 10 carbonate buffer gave the maximum absorbance and the faster reaction rate. Figure 2A shows the Tª effect on the reaction between 10 and 45 °C. As can be seen, the highest the temperature, the lowest the sensitivity. Different hypotheses were considered to explain these results: (1) NADH decomposition with temperature (but, according to bibliography [23] this requires longer reaction times), (2) enzyme degradation (that was not visually observed) and (3) the K_eq_ of the reaction inversely increases with temperature which indicates that the enzymatic reaction is exothermic. This last was tested. In the experimental conditions tested it can be deduced that:

4 $$K_{eq}={\frac{{Abs}_{eq}^2}{\left({\lbrack NAD\rbrack}_0\varepsilon_{NADH}-{Abs}_{eq}\right)\left({\lbrack Trp\rbrack}_0\varepsilon_{NADH}-{Abs}_{eq}\right)}=\left(\frac{{Abs}_{eq}}{6.3-{Abs}_{eq}}\right)^2}$$

e_NADH_ being the NADH molar absorptivity at 340 nm (6300 M^−1^ cm^−1^, experimentally obtained) and [NAD]_0_ and [Trp]_0_ the initial concentrations used for the experiments. Figure 2B, shows the fitting of the results to the Van’t Hoff Eq. (5):

5 $$ln{K}_{Eq}=-\frac{\Delta {H}^{0}}{R}\cdot \frac{1}{T}+\frac{{\Delta S}^{0}}{R}$$

From these results the ΔH^0^ ($-565\pm 34 K J/mol)$ and ΔS^0^ ($=-1.82\pm 0.10KJ/mol K$) values were obtained. These results agree with the exothermicity of the reaction and, more important, allows to predict the K_eq_ to other working temperatures.

**Fig. 3:**
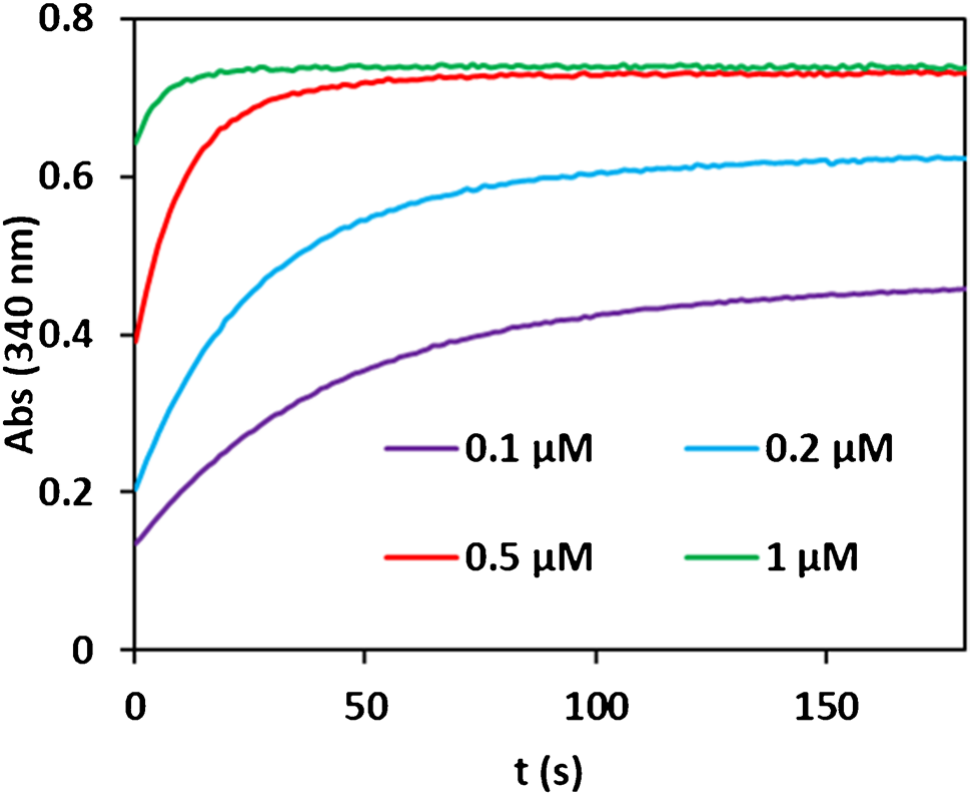
Abs=f(t) recordings obtained at different concentrations of the enzyme. [NAD] = 1.0·10^−3^ M, [Trp] = 1.0·10^−4^ M, 25ºC, pH = 10 (HCO_3_^-^/CO_3_^2-^ 0.1 M), λ = 340 nm

Interesting results were also obtained during enzyme concentration optimization. As expected, as the enzyme concentration decreases, the reaction slows down, but unexpectedly, the equilibrium constant becomes higher (Fig. 3). This effect on the K_eq_ has been previously observed in other dehydrogenase reactions by authors such as Theorell (with alcohol dehydrogenase) [24] or Alberty (with lactate dehydrogenase) [25]. The explanation is due to the NAD/NADH-enzyme complex formation. The difference between the observed or apparent Keq (K_eq,ap_) and the real Keq (K_eq,real_) can be expressed as follows:

6 $$\frac{{K}_{app}}{{K}_{real}}= \frac{{K}_{E-NAD}\left({[E]}_{0}+{K}_{E-NADH} \right)}{{K}_{E-NADH}\left({[E]}_{0}+{K}_{E-NAD} \right)}$$

K_E-NAD_ and K_E-NADH_ being the Enzyme-NAD and Enzyme-NADH dissociation constants. When the enzyme concentration ([E]_0_) is high, Eq. (6) trends to:

7 $$\frac{{K}_{app}}{{K}_{real}}= \frac{{K}_{E-NAD}}{{K}_{E-NADH}}$$

and K_eq,ap_ is independent of the enzyme concentration (as observed in Fig. 3).

Trp is also a tropane alkaloid, which is able to produce similar problems to that of Atp in human bodies, albeit less seriously. This compound appears in several food samples so its determination can also be of interest. For this reason, the analytical figures of merit for Trp were obtained. Figure S4 shows the calibration line which justifies a linear relationship from 9.0·10^−6^ (limit of quantification) to 1.0·10^−4^ M, the equation of the calibration line being ([Trp]_0_ in M):

8 $${\mathrm{Abs}}_{340}=6151\left(\pm115\right)\cdot{\left[\mathrm{Trp}\right]}_0+0.050\left(\pm0.003\right)\mathrm R^2=0.999$$

The limit of detection (from 3s_bl_) is 2.8·10^−6^ M and a 2.2% RSD for 6.0·10^−5^ M (n = 5) was measured. Considering the stoichiometry of the reaction, if 100% of Trp reacts during the process, the slope of the calibration should be the e_NADPH_, i.e., 6300 M^−1^ cm^−1^. Compared to the value obtained (8), the conversion is 98 ± 2%, indicating that all the Trp is converted to product during the enzymatic reaction under the experimental conditions found.

#### Development of a method for atropine (Atp) determination

The second step involves the Atp hydrolysis. Two procedures were tested: enzymatic hydrolysis, using tropine esterase (TEase) and chemical hydrolysis in basic media. Inadequate yield was obtained with TEase so chemical hydrolysis was optimized. 2 M NaOH was used, and different hydrolysis times were assayedFigure S5 demonstrates that extending the reaction time beyond 5 min does not enhance hydrolysis yield.

Using a hydrolysis time of 5 min and the previously optimized TRase conditions, the analytical figures of merit obtained for the determination of Atp were as follows: (i) The calibration plot (Fig. S6) was linear from 1.1·10^−5^ M (limit of quantification) to 3.0·10^−4^ M, the equation of the line being:

9 $${\mathrm{Abs}}_{340}=5080\left(\pm77\right)\cdot{\left[\mathrm{Trp}\right]}_0+0.050\left(\pm0.009\right)\mathrm R^2=0.999$$

(ii) A detection limit of 3.5·10^−6^ M and an RSD of 3.0% for 6.0·10^−5^ M (n = 5) were obtained.

Again, the comparison of the slope of the calibration line with the molar absorptivity of NADH (6300 M^−1^ cm^−1^) allows the ATP conversion during the reaction to be determined, which in this case is 81 ± 1%, which means that the yield is reasonably high.

It is very well known that NADH also present fluorescence (460 nm) under excitation at 340 nm. A complementary study was carried using this fluorescence for Atp determination. pH and buffer nature (Fig. S7) and enzyme concentration (Fig. S8) were studied, obtaining optimum conditions very similar to those of absorbance. In these conditions the analytical figures of merit were also obtained. A linear response range from 1.6·10^−7^ M (limit of quantification) to, at least, 3·10^−6^ M was found (Fig. S9), with a limit of detection of 4.7·10^−8^ M (which is very close to those given using immunoassay). As can be seen, this fluorescence allows to improve more than one order of magnitude the limit of quantification. The most significant issue with fluorescence is inner filter effect interference, which commonly occurs in real samples when working at these wavelengths (λex = 340 nm, λem = 460 nm) due to the absorption of interfering species at the excitation and/or emission wavelength of the fluorophore [26].

Finally, the method was applied to the determination of atropine in a chia seed sample. After submitting the sample to the procedure described in the “Materials and methods” section, Atp concentration under the limit of detection was found both using the absorption and the fluorescence method. A dopped sample was then prepared containing 0.87 mg/g of Atp. The determination was carried using molecular absorption (a high matrix effect was observed using fluorescence). A 0.84 ± 0.05 mg/g (n = 3) was obtained, indicating that a satisfactory recovery of 96.3 ± 6.0%.

### Scopolamine determination with tropinone reductase

**Fig. 4:**
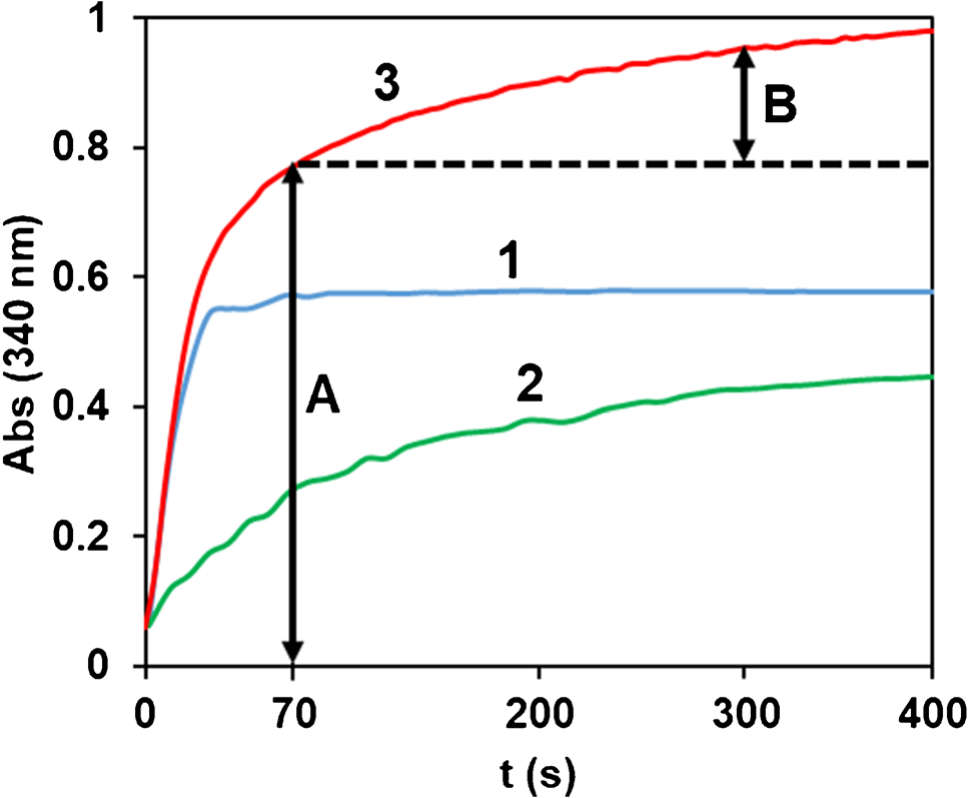
Abs_340nm_=f(t) representations obtained at λ = 340 nm corresponding to: 1) Atp (1.0·10^−4^M); 2) Scp (1.0·10^−4^M); 3) Atp (1.0·10^−4^M) +Scp (1.0·10^−4^M). In curve 3, line A shows the Abs_70_ (absorbance at 70 s) and line B shows the Abs_300_–Abs_70_ (absorbance difference between t=300 s and t=70 s). Experimental conditions: [NAD] =1.0·10^−3^ M, 25ºC, pH = 10 (HCO_3_^-^/CO_3_^2-^ 0.1 M)

Figure 4 compares the Abs = f(t) profile obtained for Atp and Scopolamine (Scp) in the experimental conditions optimized for Atp. As can be seen, the kinetics of the reaction for Scp is slower than that for Atp. Using the same experimental conditions optimized for Atp, a calibration study was carried out for Scp (Fig. S10A). Not only was the time required to reach the equilibrium much longer than for Atp, but the slope of the calibration line was more than three times lower, indicating a 25% conversion of Scp to products under these conditions.

**Fig. 5:**
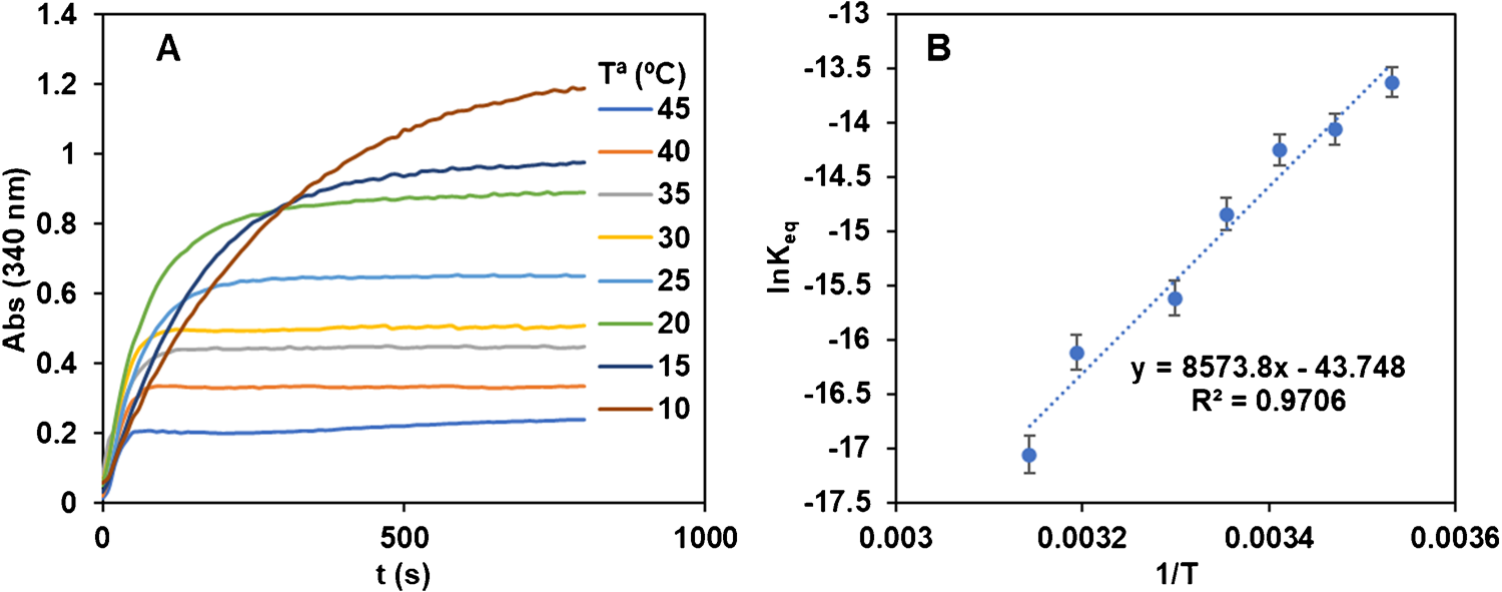
**A)** Abs_340nm_=f(t) recordings obtained at different working temperatures. [NAD] =1.0·10^−3^ M, [Scp] = 3.0·10^−4^ M, [TRase] = 0.5 mM, pH = 10 (HCO_3_^-^/CO_3_^2-^ 0.1 M); **B)** Variation of the ln_Keq_=f(1/T) derived from values of figure A

Additional studies were carried out to improve sensitivity and kinetics for Scp. No improvements were observed by changing pH or enzyme concentration. The most significant results observed were those obtained when temperature was studied. As with Atp, Fig. 5A shows that the higher the temperature the lower the Abs_eq_ but the faster the reaction. However, the effect of the temperature was more pronounced than for Atp. As shown in Fig. 2A, temperatures below than 25 °C did not modify the K_eq_ of the reaction for Atp, but in the case of Scp lower temperatures still increased the K_eq_ of the reaction. The results in Fig. 5A were again treated with Eqs. (4) and (5) (Fig. 5B)*.* From these results, the values DH^0^ ($-713\pm 34 K J/mol)$ and DS^0^ ($=-0.364\pm 0.10KJ/mol K$) were obtained, which again agree with the exothermicity of the reaction and the higher effect of temperature in K_eq_.

Then, using 15 °C as the optimum temperature, the analytical figures of merit for Scp were obtained (Fig. S10B). The calibration line was linear from 1.2·10^−5^ (limit of quantification) to 3.0·10^−4^ M, the limit of detection was 3.6·10^−6^ M, the slope of the calibration line was 4912 ± 80 (78 ± 1% transformation) and a RSD of 3.2% for 6.0·10^−4^ M (n = 5). These figures of merit are very similar those of Atp, indicating that this method is suitable for the determination of Scp. This method was also applied to the determination of Scp in the same sampled of Chia seeds spiked with 3.03 mg/g Scp. A 2.86 ± 0.07 mg/g (n = 3) was obtained, indicating that a similar recovery (94.3 ± 2.2%) as for the determination of Atp.

Unlike Trp, Sci is not a compound of analytical interest. However, a calibration line of Sci was run at the optimum conditions for Scp (i.e., 15 °C) to obtain the hydrolysis yield. Figure S11 shows that the slope of the calibration line was 5090 indicating that the oxidation yield of Sci is 81 ± 2%. Comparing this slope with that of the Scp calibration line (4912 ± 80) shows that a good Scp yield is approximately 97 ± 2%.

### Simultaneous determination of Scp and Atp

Given the results described above, it is possible to determine Atp and Scp simultaneously in a sample. Two alternative procedures were considered.

Firstly, the optimum working temperature for both compounds is different and the temperature effect of T in the K_eq_ is also different. It is therefore possible to measure mixtures at two different temperatures. According to this procedure, calibration lines of Scp and Atp were carried out at 35° and 15° and then mixtures were measured at these two temperatures. Inconsistent results were obtained, so this methodology was discarded.

Secondly, according to Fig. 5, Atp oxidation is faster than Scp at 25 °C. It would be possible to distinguish between these two compounds by taking measurements at two (or more) different times. This method gave better results and was use throughout.

When measuring Atp, the time required to obtain the Abs_eq_ (t_eq_) depends on the Atp concentration: the higher the concentration the higher the t_eq_; the maximum value is 60 s, corresponding to the maximum At concentration of the calibration line. If the enzymatic reaction is carried out in a mixture containing Atp + Scp, the NADH formed at reaction times greater than 60 s should be due to Scp only, so that any variation in absorbance from this time should allow the determination of Scp without interference from Atp. The absorbance at 300 s (Abs_m,300_) and 70 s (Abs_m,70_) of a mixture is then measured, and Abs_m,300_ − Abs_m,70_ was used to determine Scp (Fig. 

S12). Then, the absorbance at 300 s due to Scp (Abs_s,300_) is calculated from the corresponding calibration line, and Abs_m,300_ − Abs_s,300_ is used for Atp determination.

To test the validity of this procedure, three synthetic samples containing Atp and Scp were prepared, and the contents of these samples were measured. Error standards (RE) of 5.6% for Atp and 7.0% for Scp calculated according to (10) were obtained (see Table S7).

10 $$RE=100\sqrt{\frac{\sum_{i}{\left({C}_{m}-{C}_{r}\right)}^{2}}{\sum_{i}{\left({C}_{r}\right)}^{2}}}$$

Finally, the aforementioned sample of chia seeds previously was spiked with Atp (0.29% w/w) and Scp (0.30% w/w) and subjected to the previously described method. The results obtained (n = 3) were 0.27 ± 0.01% (recovery 92 ± 3%) for Atp, and 0.28 ± 0.01% (recovery 94 ± 2%) for Scp. These results indicate that a quantitative determination of both compounds is possible using this method.

### Comparison with other methods

Table S8 compares the results obtained in this paper with the obtained by classical techniques. Despite not obtaining limits of detection and quantification comparable to those of mass spectrometry, this methodology offers other advantages, such as (i) speed and simplicity suitable for screening methods and (ii) the possibility of analyzing and differentiating atropine and scopolamine together.

## Conclusions

This paper demonstrates that the enzyme TRase catalyzes the oxidation of both tropine and scopine according to a compulsory order ternary complex kinetic model, which has allowed to characterize the kinetic constants of both compounds. It is important to note the negative effect of the temperature on the K_eq_ of the reaction of both substrates, as well as the effect of enzyme concentration on K_eq_.

From that, methodologies have been developed for the determination of atropine (Atp) and scopolamine by means of the NADH signal at 340 nm generated in the TRase reaction. Moreover, the difference in kinetics of both analytes allows their simultaneous determination. Despite the analytical figures of merit of this method for tropane alkaloids are slightly worse than those reported using other methods, this is an important analytical advantage over immunoassay-based determination methods and other classical methodologies in which the selectivity of the immunoreagent only allows the determination of atropine or scopolamine.

## Supplementary Information

Below is the link to the electronic supplementary material.

ESM 1 (DOCX 366 KB)

## Data Availability

All data and materials are included in the manuscript and in the supplementary material.
