# Supplementary material for: Kinetic and analytical characterization of a new tropinone oxidase enzyme and its application to the simultaneous determination of the tropane alkaloids atropine and scopolamine
Source: Anal Bioanal Chem. 2025 Apr 10;417(14):3169–76. doi: 10.1007/s00216-025-05856-6 (PMC12103354; doi:10.1007/s00216-025-05856-6)
Supplement: Supplementary file 1 — (DOCX 366 KB) [file 216_2025_5856_MOESM1_ESM.docx]

**Supplementary Material**

**Kinetic and analytical characterization of a new tropinone oxidase enzyme and its application to the simultaneous determination of the tropane alkaloids atropine and scopolamine**

Mario Domínguez, Susana de Marcos, Javier Galbán*

Analytical Chemistry Department, University of Zaragoza and Instituto de Nanociencia y Materiales de Aragón (INMA), CSIC-Universidad de Zaragoza. Zaragoza 50009, Spain

* Corresponding author: jgalban@unizar.es

**Index**

**Section S1. Kinetic mechanism for tropine (Trp)…………….……… 2**

**S1.1. Elucidation of the kinetic mechanism …………..……. 2**

Table S1……………..…………………..……………………. 2

Figure S1 ……..…………………………..…………………… 2

Table S2……………..…………………..……………………. 3

**S1.2. Approximate calculation of the kinetic constants ….. 3**

**S1.3. Precise calculation of the kinetic constants …………. 3**

Table S3 ………….…………………....……………………... 4

**Section S2. Kinetic mechanism for scopine (Sci)…………….………. 4**

**S2.1. Elucidation of the kinetic mechanism …………..……… 4**

Table S4……………..…………………..……………………… 4

Figure S2 ……..…………………………..……………………. 5

Table S5 ………….…………………....………………………. 5

**S2.2. Approximate calculation of the kinetic constants ……. 5**

**S2.3. Precise calculation of the kinetic constants …………… 6**

Table S6 ………….…………………....………………………. 6

**Section S3. Optimization of the analytical method…………….……… 6**

Figure S3 ……..…………………………..…………………….. 6

Figure S4 ……..…………………………..……………………. 7

Figure S5 ……..…………………………..……………………. 7

Figure S6 ……..…………………………..……………………. 7

Figure S7 ……..…………………………..……………………. 8

Figure S8 ……..…………………………..……………………. 8

Figure S9 ……..…………………………..……………………. 8

Figure S10 ……..………………..………..…………………… 9

Figure S11 ……..…………………..……..…………………… 9

Figure S12 ……..…………………..……..…………………… 9

Table S7 ……..……………………..……..…………………… 10

**Section S4. Comparison with other methods …………………….……… 10**

**Section S1. Kinetic mechanism for Tropine (Trp)**

**S1.1. Elucidation of the kinetic mechanism**

Firstly, for the sake of clarity, equations (1), (2) and (3) of the main manuscript will be reproduced here as (*S1*), (*S2*) and (*S3*) respectively. Note that (S1) and (S3) refer to the COTC mechanism and (S2) to the ping-pong mechanism.

$$\frac{1}{V_{0}}=\left( \frac{K_{m,Trp}}{k_{cat}{[TRase]}_{0}}+\frac{K_{i,NAD}K_{m,Trp}}{k_{cat}{[TRase]}_{0}{[NAD]}_{0}} \right)\frac{1}{{[Trp]}_{0}}+\left( \frac{1}{k_{cat}{[TRase]}_{0}}+\frac{K_{m,NAD}}{k_{cat}{[TRase]}_{0}{[NAD]}_{0}} \right) (S1)$$

$$\frac{1}{V_{0}}=\left( \frac{K_{m,Trp}}{k_{cat}{[TRase]}_{0}} \right)\frac{1}{\left[ Trp \right]_{0}} + \left( \frac{K_{m,NAD}}{k_{cat}{[TRase]}_{0}\left[ NAD \right]_{0}}+\frac{1}{k_{cat}{[TRase]}_{0}} \right) \left( S2 \right)$$

$$V_{0}=\frac{k_{cat}{[TRase]}_{0}\left[ NAD \right]}{K_{a}\left[ NAD \right]+K_{b}\left[ Trp \right]+\left[ Trp \right]\left[ NAD \right]+K_{iA}K_{b}} (S3)$$

Table S1 shows the initial velocities (s^-1^) obtained using six different NAD concentrations and six different tropine concentrations. The experimental conditions were: λ=340 nm, [TRase]=0.50 μM, pH=10 (carbonate buffer) and 25ºC.

**Table S1:** initial rates of the Trp/TRase reaction

| **NAD,M Trp, M** | **5.0·10^-5^** | **1.0·10^-4^** | **3.0·10^-4^** | **6.0·10^-4^** | **1.0·10^-3^** | **2.0·10^-3^** |
| --- | --- | --- | --- | --- | --- | --- |
| **1.0·10^-5^** | 5.08·10^-4^ | 6.22·10^-4^ | 2.16·10^-3^ | 4.40·10^-3^ | 6.76·10^-3^ | 5.24·10^-3^ |
| **3.0·10^-5^** | 7.29·10^-4^ | 2.61·10^-3^ | 7.11·10^-3^ | 1.51·10^-2^ | 1.70·10^-2^ |  |
| **6.0·10^-5^** | 2.06·10^-3^ | 7.61·10^-3^ | 1.29·10^-2^ | 2.38·10^-2^ | 2.73·10^-2^ | 3.04·10^-2^ |
| **1.0·10^-4^** | 3.26·10^-3^ | 1.07·10^-2^ | 2.34·10^-2^ | 4.05·10^-2^ | 4.34·10^-2^ | 4.65·10^-2^ |
| **3.0·10^-4^** | 5.32·10^-3^ | 2.25·10^-2^ | 3.83·10^-2^ | 7.28·10^-2^ | 7.93·10^-2^ | 1.05·10^-1^ |
| **6.0·10^-4^** | 1.12·10^-2^ | 2.06·10^-2^ | 4.85·10^-2^ | 8.47·10^-2^ | 1.08·10^-1^ | 1.26·10^-1^ |

From these results, the inverse of the initial velocities (1/V_0_) was calculated, and the representation 1/V_0_=f([Trp]) was obtained for each [NAD] concentration (Lineweaver-Burk formalism). The results are shown in Figure S1. The slope and the intercept of each plot were obtained; values are given in Table S2.


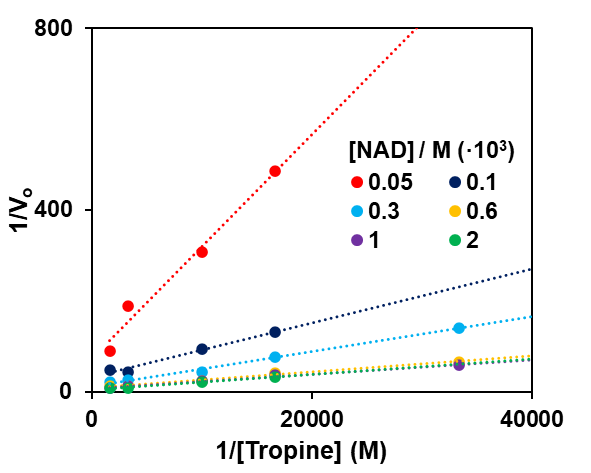


**Figure S1.** 1/V_0_=f(1/[Trp]_0_) for 6 different concentrations of NAD.

**Table S2.** Slope and intercept for each concentration of NAD

| **[NAD], M** | **b_1_=slope** | **b_0_=intercept** |
| --- | --- | --- |
| **5.0·10^-5^** | 2.4683·10^-2^ | 72.280 |
| **1.0·10^-4^** | 5.9422·10^-3^ | 32.347 |
| **3.0·10^-4^** | 3.8545·10^-3^ | 11.494 |
| **6.0·10^-4^** | 1.7626·10^-3^ | 8.764 |
| **1.0·10^-3^** | 1.5712·10^-3^ | 7.625 |
| **2.0·10^-3^** | 1.6972·10^-3^ | 4.521 |

When the slopes (b_1_) and intercepts (b_0_) given in Tables S2 are plotted as a function of 1/[NAD]_0_, a linear (least squares) relationship are obtained according to the following equations:

$b_{0}=0.0034 \frac{1}{{[NAD]}_{0}}+2.119 R^{2}=0.992 (S4)$

$b_{1}=4.72\cdot{10}^{-7} \frac{1}{{[NAD]}_{0}}+1.19\cdot{10}^{-3} R^{2}=0.991 (S5)$

Equation (S1) justifies that if the kinetic follows the COTC, the intercepts (b_0_) will linearly depend on the [NAD] concentration used. Conversely, if the kinetic follows the ping-pong mechanism (S2), b_0_ should be independent of [NAD]_0_ concentration. As shown in (S4), the kinetic follows the COTC mechanism.

**S1.2. Approximation of kinetic constants**

The next step will be the approximate calculation of the constants that appear in equation (S2). According to it:

$$b_{0}=\frac{K_{m,NAD}}{k_{cat}{\left[ TRase \right]_{0}\left[ NAD \right]}_{0}}+\frac{1}{k_{cat}\left[ TRase \right]_{0}}=\left( \frac{K_{m,NAD}}{V_{0}} \right)\frac{1}{\left[ NAD \right]_{0}}+\frac{1}{k_{cat}\left[ TRase \right]_{0}} \left( S6 \right)$$

Comparing (S4) and (S5) allows to calculate K_m,NAD_ and k_cat_ (bear in mind that the [TRase]= 0. 50 μM).

$$k_{cat}=9.42*{10}^{5} s^{-1} K_{m,NAD}=1.6043\cdot{10}^{-3} M (S7)$$

Considering again (S2):

$$b_{1}=\frac{K_{m,Trp}}{k_{cat}\left[ TRase \right]_{0}}+\left( \frac{K_{i,NAD}K_{m,Trp}}{k_{cat}\left[ TRase \right]_{0}} \right)\frac{1}{{[NAD]}_{0}} \left( S8 \right)$$

And comparing (S5) with (S8) and taking into account (S7):

$$K_{m,Trp}=5.6094\cdot{10}^{-4} M K_{i,NAD}=1.3889\cdot{10}^{-4} M (S9)$$

**S1.3. Precise calculation of the kinetic constants**

In this case, the calculation of these constants using the Lineweaver-Burk gives more weight to the smaller V_0_ values which are less precise spectrophotometrically. For this reason, a more precise calculation of these constants has been made using equation (S3). This equation can be rewritten as:

$$v=\frac{\alpha\left[ Trp \right]}{\beta\left[ Trp \right]+\gamma} (S10)$$

Where:

$\alpha=k_{cat}\left[ TRase \right]_{0}\left[ NAD \right] \beta=K_{m,Trp}+\left[ NAD \right] \gamma=K_{m,NAD}\left[ NAD \right]+K_{i,NAD}K_{m,Trp} (S11)$

Using the values previously obtained (S(7) and S(9)), the approximate values of α, β and γ were calculated for each concentration of NAD. These values feed the Solver™ routine of an Excel™ spreadsheet, which allows these values to be calculated more precisely (table S3).

**Table S3.** α, β, and γ values obtained for each concentration of NAD using the Solver^TM^ routine

| **[NAD], M** | **α** | **β** | **γ** |
| --- | --- | --- | --- |
| **5.0·10^-5^** | 2.1958·10^-5^ | 1.7151·10^-3^ | 4.5696·10^-7^ |
| **1.0·10^-4^** | 4.7185·10^-5^ | 1.7043·10^-3^ | 2.4406·10^-7^ |
| **3.0·10^-4^** | 1.4156·10^-4^ | 1.9043·10^-3^ | 3.9111·10^-7^ |
| **6.0·10^-4^** | 2.8311·10^-4^ | 2.2043·10^-3^ | 5.4678·10^-7^ |
| **1.0·10^-3^** | 4.7185·10^-4^ | 2.6043·10^-3^ | 7.8377·10^-7^ |
| **2.0·10^-3^** | 7.8418·10^-4^ | 3.9683·10^-3^ | 1.2075·10^-6^ |

With these parameters optimized, the constants are recalculated by fitting α, β and γ to the NAD concentration using the least squares method. The following final values are obtained:

$$\boldsymbol{k}_{\boldsymbol{cat}}\boldsymbol{=6.2295}\left( \boldsymbol{\pm0.0050} \right)\boldsymbol{\cdot}\boldsymbol{10}^{\boldsymbol{5}} \boldsymbol{s}^{\boldsymbol{-1}} \boldsymbol{K}_{\boldsymbol{m}\boldsymbol{,}\boldsymbol{NAD}}\boldsymbol{=1.62624}\left( \boldsymbol{\pm0.00038} \right)\boldsymbol{\cdot}\boldsymbol{10}^{\boldsymbol{-3}}\boldsymbol{M. (S}\boldsymbol{12)}$$

$$\boldsymbol{K}_{\boldsymbol{m}\boldsymbol{,}\boldsymbol{Trp}}\boldsymbol{=5.01}\left( \boldsymbol{\pm0.27} \right)\boldsymbol{\cdot}\boldsymbol{10}^{\boldsymbol{-4}} \boldsymbol{M} \boldsymbol{K}_{\boldsymbol{i}\boldsymbol{,}\boldsymbol{NAD}}\boldsymbol{=4.67}\left( \boldsymbol{\pm0.61} \right)\boldsymbol{\cdot}\boldsymbol{10}^{\boldsymbol{-4}} \boldsymbol{M (S}\boldsymbol{13)}$$

**Section S2. Kinetic mechanism for Scopine (Sci)**

**S2.1. Elucidation of the kinetic mechanism**

Table S4 shows the Initial velocities (s^-1^) obtained using six different NAD concentrations and six different scopine concentrations. The experimental conditions were: λ=340 nm, [TRase]=0.5 μM, pH=10 (carbonate buffer) and 15ºC.

**Table S4:** initial rates of the Sci/TRase reaction

| **NAD,M Sci, M** | **5.0·10^-5^** | **1.0·10^-4^** | **3.0·10^-4^** | **6.0·10^-4^** | **1.0·10^-3^** | **2.0·10^-3^** |
| --- | --- | --- | --- | --- | --- | --- |
| **3.0·10^-5^** | — | 3.971·10^-5^ | 3.114·10^-4^ | 8.462·10^-4^ | 1.510·10^-3^ | 1.801·10^-3^ |
| **6.0·10^-5^** | 5.181·10^-5^ | 9.161·10^-5^ | 5.354·10^-4^ | 1.129·10^-3^ | 2.036·10^-3^ | 2.667·10^-3^ |
| **1.0·10^-4^** | 2.705·10^-4^ | 1.807·10^-4^ | 9.893·10^-4^ | 2.221·10^-3^ | 3.475·10^-3^ | 3.533·10^-3^ |
| **3.0·10^-4^** | 6.888·10^-4^ | 1.163·10^-3^ | 3.876·10^-3^ | 4.366·10^-3^ | 6.528·10^-3^ | 6.055·10^-3^ |
| **6.0·10^-4^** | 1.176·10^-3^ | 1.876·10^-3^ | 5.140·10^-3^ | 5.838·10^-3^ | 6.867·10^-3^ | 7.847·10^-3^ |
| **1.0·10^-3^** | 1.698·10^-3^ | 3.013·10^-3^ | 5.885·10^-3^ | 6.253·10^-3^ | 7.363·10^-3^ | 8.415·10^-3^ |

Figure S2 shows the 1/V_0_=f([Sci]) representation for each [NAD]_0_ concentration. The slope and intercept of each representation were obtained and the values are given in table S5.

**
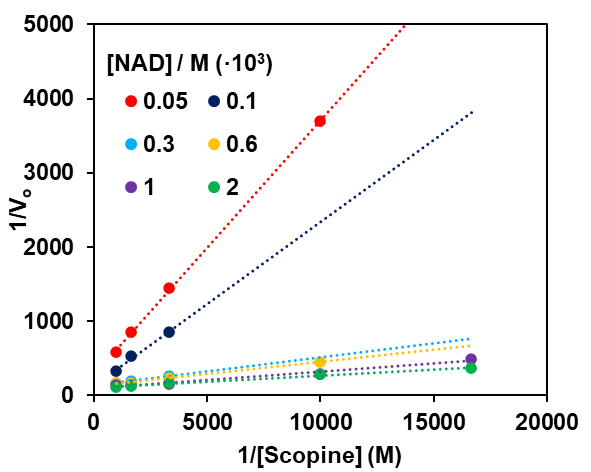
**

**Figure S2.** 1/V_0_=f(1/[Sci]_0_) for 6 different concentrations of NAD.

**Table S5.** Slope and intercept for each concentration of NAD

| **[NAD], M** | **b_1_=slope** | **b_0_=intercept** |
| --- | --- | --- |
| **5.0·10^-5^** | 3.4316·10^-1^ | 274.42 |
| **1.0·10^-4^** | 2.2038·10^-1^ | 134.06 |
| **3.0·10^-4^** | 3.7813·10^-2^ | 131.87 |
| **6.0·10^-4^** | 3.2804·10^-2^ | 121.41 |
| **1.0·10^-3^** | 2.2445·10^-2^ | 96.047 |
| **2.0·10^-3^** | 1.6596·10^-2^ | 105.46 |

These results lead to the following equations for the slopes (b_1_) and intercepts (b_0_), which demonstrate the fulfilment of the COTC mechanism.

$b_{0}=0.0081 \frac{1}{{[NAD]}_{0}}+94.7 R^{2}=0.89 (S14)$

$b_{1}=1.76\cdot{10}^{-5} \frac{1}{{[NAD]}_{0}}+4.62\cdot{10}^{-3} R^{2}=0.97 (S15)$

**S2.2. Approximation of kinetic constants**

Following the same procedure as in Trp the following constants were obtained:

$$k_{cat}=2.11\cdot{10}^{4} s^{-1} K_{m,NAD}=8.55\cdot{10}^{-5} M (S16)$$

$$K_{m,Sci}=5.67\cdot{10}^{-5} K_{i,NAD}=3.83\cdot{10}^{-4} (S17)$$

**S2.3. Precise calculation of the kinetic constants**

Using the Solver™ routine, the more reliable values were obtained (Table S6), and the kinetics constant calculated.

**Table S6.** α, β, and γ values obtained for each concentration of NAD using the Solver^TM^ routine

| [**NAD], M** | **α** | **β** | **g** |
| --- | --- | --- | --- |
| **5.0·10^-5^** | 5.2784·10^-7^ | 1.3551·10^-4^ | 1.8913·10^-7^ |
| **1.0·10^-4^** | 1.0557·10^-6^ | 1.8551·10^-4^ | 1.9156·10^-7^ |
| **3.0·10^-4^** | 3.1648·10^-6^ | 3.8551·10^-4^ | 1.5773·10^-7^ |
| **6.0·10^-4^** | 6.3341·10^-6^ | 6.8551·10^-4^ | 2.1596·10^-7^ |
| **1.0·10^-3^** | 1.0557·10^-5^ | 1.0855·10^-3^ | 2.1420·10^-7^ |
| **2.0·10^-3^** | 2.1114·10^-5^ | 2.0855·10^-3^ | 2.8426·10^-7^ |

$$\boldsymbol{k}_{\boldsymbol{cat}}\boldsymbol{=1.391}\left( \boldsymbol{\pm0.002} \right)\boldsymbol{\cdot}\boldsymbol{10}^{\boldsymbol{4}} \boldsymbol{s}^{\boldsymbol{-1}} \boldsymbol{K}_{\boldsymbol{m}\boldsymbol{,}\boldsymbol{NAD}}\boldsymbol{=8.6}\left( \boldsymbol{\pm0.1} \right)\boldsymbol{\cdot}\boldsymbol{10}^{\boldsymbol{-5}}\boldsymbol{M. (S}\boldsymbol{18)}$$

$$\boldsymbol{K}_{\boldsymbol{m,Sci}}\boldsymbol{=5}\left( \boldsymbol{\pm1} \right)\boldsymbol{\cdot}\boldsymbol{10}^{\boldsymbol{-5}}\boldsymbol{M}\boldsymbol{K}_{\boldsymbol{i,NAD}}\boldsymbol{=3.3}\left( \boldsymbol{\pm0.8} \right)\boldsymbol{\cdot}\boldsymbol{10}^{\boldsymbol{-3}}\boldsymbol{M (S}\boldsymbol{19)}$$

**Section S3.- Optimization of the analytical method**


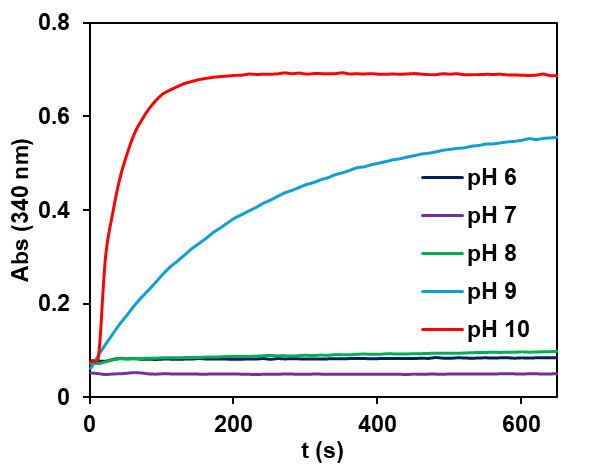


**Figure S3:** Optimization of the pH and buffer for the determination of Trp. Abs=f(t) at 340 nm obtained for different pH and buffers. The experimental conditions used were [NAD] = 1.0·10^-3^ M, [Trp] = 1.0·10^-4^ M, [TRase] = 0.5 μM, Tª = 30ºC. 0.1M NaH_2_PO_4_/ Na_2_HPO_4_ buffer was used for pH 6, 7 and 8; 0.1 NaHCO_3_/Na_2_CO_3_ buffer was used for pH 10.


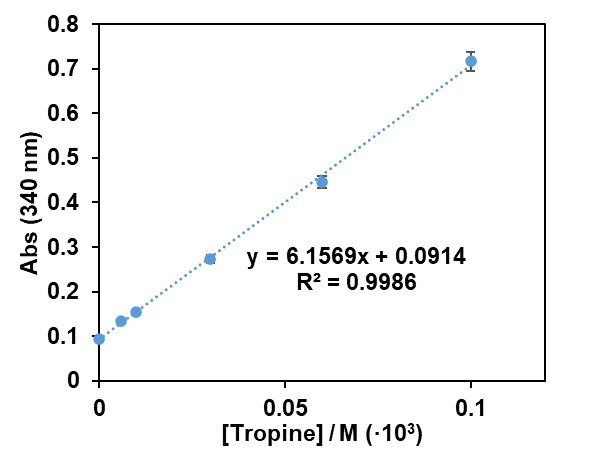


**Figure S4:** Calibration line obtained for Trp. [NAD] = 1.0·10^-3^ M [TRase] = 0.5 μM, Tª = 25ºC, pH = 10 0.1M NaHCO_3_/Na_2_CO_3_. λ=340 nm.


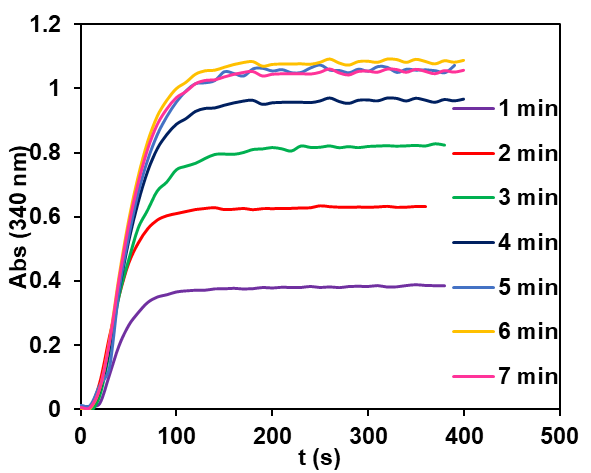


**Figure S5:** Optimization of the hydrolysis time of Atp. Abs=f(t) recorders (340 nm) obtained with different hydrolysis time (2M NaOH). [NAD] = 1.0·10^-3^ M, [Atp] = 2.0·10^-4^ M, [TRase] = 0.5 μM, Tª = 25ºC, pH = 10 0.1M NaHCO_3_/Na_2_CO_3_.


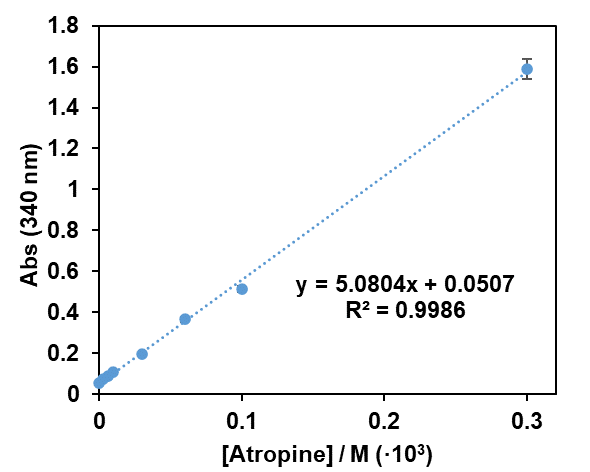


**Figure S6:** Calibration line obtained for Atp. 5 min hydrolysis 2M NaOH. [NAD]=1.0·10^-3^ M [TRase] = 0.5 μM, Tª = 25ºC, pH = 10 0.1M NaHCO_3_/Na_2_CO_3_. λ=340 nm.


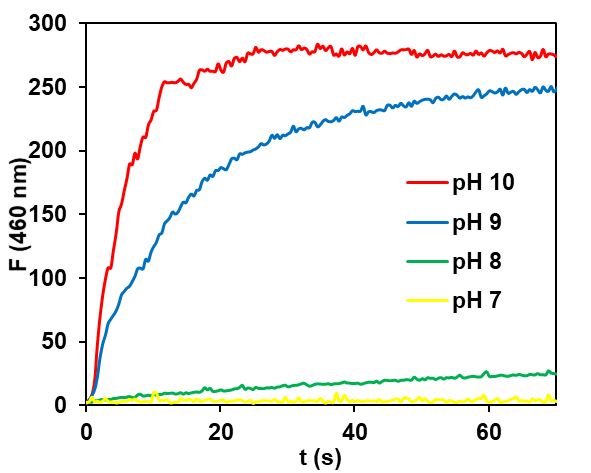


**Figure S7:** Fluorescence=f(t) (lex=340 nm and λem= 460 nm) obtained for different pH and buffers. The experimental conditions used were [NAD] = 1.0·10^-3^ M, [Atp] =1.0·10^-4^ M, [TRase] = 0.5 μM, Tª = 30ºC. 0.1M NaH_2_PO_4_/ Na_2_HPO_4_ buffer was used for pH 7 and 8; 0.1 M NaHCO_3_/Na_2_CO_3_ buffer was used for pH 10.


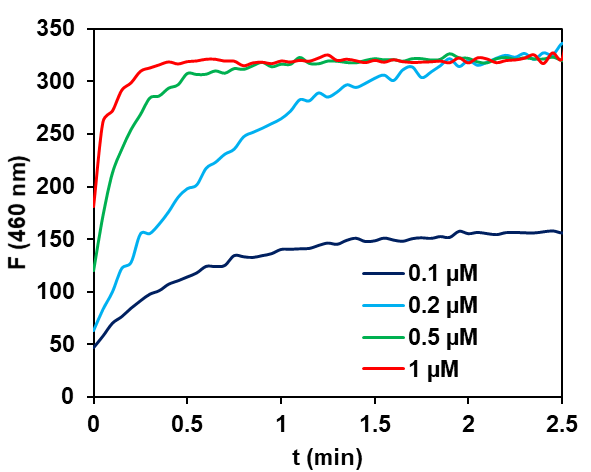


**Figure S8:** Fluorescence=f(t) (λex=340 nm and λem= 460 nm) obtained for different [TRase]. The experimental conditions used were: [NAD] = 1.0·10^-3^ M, [Atp] = 1.0·10^-4^ M, Tª = 25ºC and pH=10 0.1M NaH_2_PO_4_/ Na_2_HPO_4_ buffer.


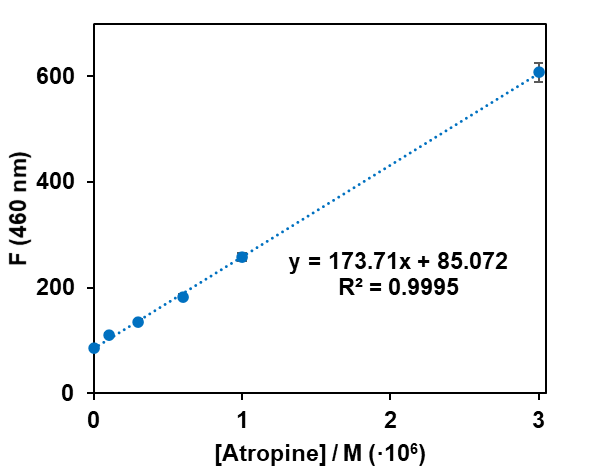


**Figure S9:** Calibration line obtained for Atp from fluorescence measurements ((λex=340 nm, λ_em_=460 nm). 5 min hydrolysis 2M NaOH, [NAD] = 1.0·10^-3^ M, [TRase] = 0.5 μM, Tª = 25ºC, pH = 10 0.1M NaHCO_3_/Na_2_CO_3_.


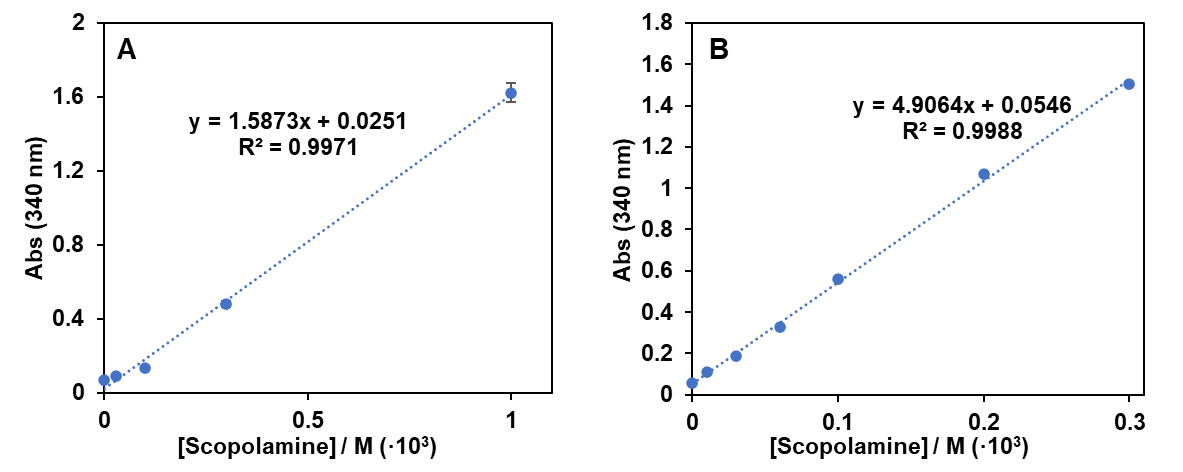


**Figure S10:** Calibration lines obtained for Scp at two different temperatures: **A)** Tª = 25ºC; **B)** Tª = 15ºC. In both cases: 5 min hydrolysis 2M NaOH, [NAD] = 1.0·10^-3^ M, [TRase] = 0.5 μM, pH =10 0.1M NaHCO_3_/Na_2_CO_3_.

**
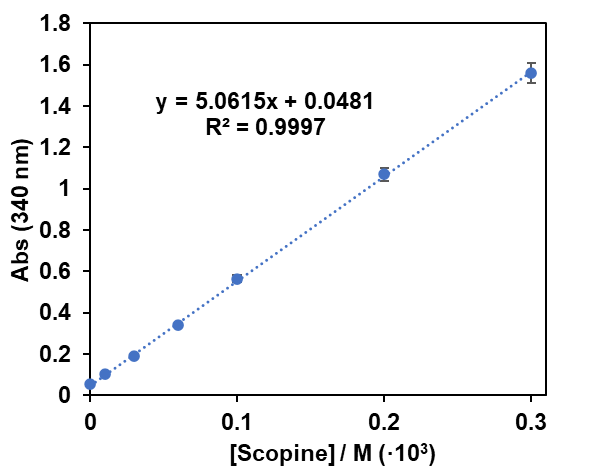
**

**Figure S11:** Calibration lines obtained for Sci. [NAD] = 1.0·10^-3^ M [TRase] = 0.5 μM, pH = 10 0.1M, Tª = 15ºC, NaHCO_3_/Na_2_CO_3_ (λ=340 nm).


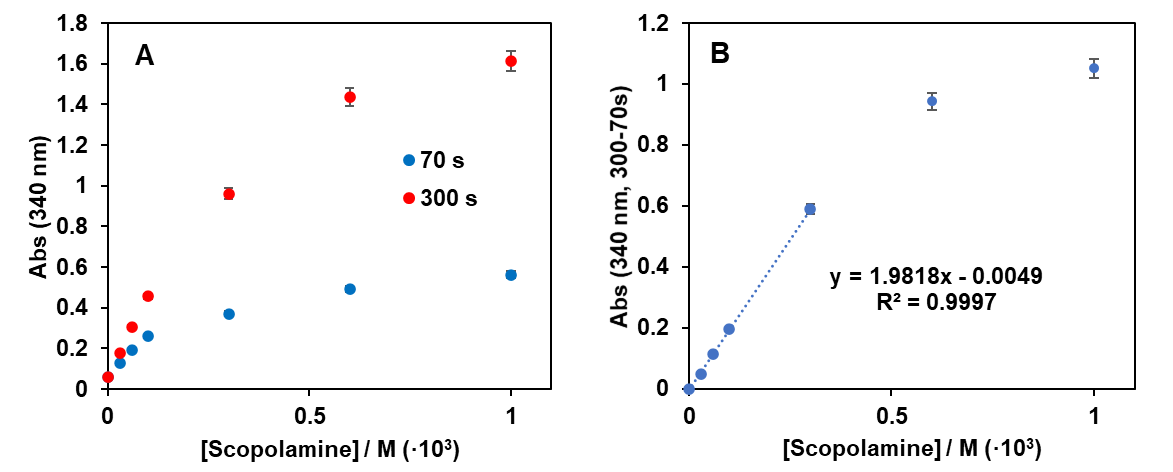


**Figura S.12:** Calibration lines obtained for Scp. **A)** at two reaction times: 300s and 70s. **B)** Difference between Abs_300_-Abs_70_. In both cases: 5 min. hydrolysis 2M NaOH. [NAD] = 1.0·10^-3^ M [TRase] = 0.5 mM, pH = 10 0.1M NaHCO_3_/Na_2_CO_3_. Tª = 25ºC. l=340 nm.

**Table S7.** Application of the method to the simultaneous determination of Atp and Scp in synthetic sample

|  | **Atp, mM** | | **Scp, mM** | |
| --- | --- | --- | --- | --- |
| **Sample** | Added | Found | Added | Found |
| **1** | 0.030 | 0.026 | 0.100 | 0.090 |
| **2** | 0.100 | 0.097 | 0.100 | 0.093 |
| **3** | 0.100 | 0.106 | 0.300 | 0.320 |

**Section S4. Comparison with other methods**

**Table S8**. Limit of detection of the most recently published methods based on classical separation techniques and those of the proposed method

| Method | Compound | LoD (mg/mL) | Reference |
| --- | --- | --- | --- |
| UV-vis absorbance | Atropine | 1.0 | This paper |
| UV-vis absorbance | Scopolamine | 1.1 | This paper |
| Fl. absorbance | Atropine | 0.013 | This paper |
| GC-MS | Atropine  Scopolamine | 0.0002 | [1] |
| GC-MS | Scopolamine | 0.005 | [2] |
| HPLC-MS/MS | Atropine  +  Scopolamine | 5·10^-5^ | [3] |
| HPLC-UV | Atropine | 0.025 | [4] |
| CE-ECL | Atropine  Scopolamine | LoD_Atr_ = 0.0046  LoD_Scp_ = 0.06 | [5] |
| CE-C^4^D | Scopolamine | 0.85 | [6] |

**References**

[1] P. Ondra, K. Zedníková, I. Válka, Detection and determination of abused hallucinogens in biological material, in: Neuroendocrinology Letters, 2006.

[2] A. Namera, M. Yashiki, Y. Hirose, S. Yamaji, T. Tani, T. Kojima, Quantitative analysis of tropane alkaloids in biological materials by gas chromatography-mass spectrometry, Forensic Sci Int 130 (2002). https://doi.org/10.1016/S0379-0738(02)00302-X.

[3] G. Koželj, L. Perharič, L. Stanovnik, H. Prosen, Simple validated LC-MS/MS method for the determination of atropine and scopolamine in plasma for clinical and forensic toxicological purposes, J Pharm Biomed Anal 96 (2014). https://doi.org/10.1016/j.jpba.2014.03.037.

[4] O. Rbeida, B. Christiaens, P. Hubert, D. Lubda, K.S. Boos, J. Crommen, P. Chiap, Integrated on-line sample clean-up using cation exchange restricted access sorbent for the LC determination of atropine in human plasma coupled to UV detection, J Pharm Biomed Anal 36 (2005). https://doi.org/10.1016/j.jpba.2004.08.007.

[5] J. Li, Y. Chun, H. Ju, Simultaneous electrochemiluminescence detection of anisodamine, atropine, and scopolamine in Flos daturae by capillary electrophoresis using β-cyclodextrin as additive, Electroanalysis 19 (2007). https://doi.org/10.1002/elan.200703903.

[6] M.M.A.C. Ribeiro, D.N. Barreto, J. Flávio da S. Petruci, E.M. Richter, Simultaneous determination of scopolamine and butylscopolamine in pharmaceutical and beverage samples by capillary zone electrophoresis, Microchemical Journal 172 (2022). https://doi.org/10.1016/j.microc.2021.106985.
